# Synthetic biology promotes the capture of CO2 to produce fatty acid derivatives in microbial cell factories

**DOI:** 10.1186/s40643-022-00615-2

**Published:** 2022-12-05

**Authors:** Xiaofang Liu, Hangyu Luo, Dayong Yu, Jinyu Tan, Junfa Yuan, Hu Li

**Affiliations:** 1 Guizhou Provincial Key Laboratory for Rare Animal and Economic Insects of the Mountainous Region, College of Biology and Environmental Engineering, Guiyang University, Guiyang, Guizhou China; 2 State Key Laboratory Breeding Base of Green Pesticide & Agricultural Bioengineering, Key Laboratory of Green Pesticide & Agricultural Bioengineering, Ministry of Education, State-Local Joint Laboratory for Comprehensive Utilization of Biomass, Center for Research & Development of Fine Chemicals, Guizhou University, Guiyang, Guizhou China

**Keywords:** Microorganisms, Carbon capture, Fatty acid derivatives, Biofuels, Synthetic biology

## Abstract

Environmental problems such as greenhouse effect, the consumption of fossil energy, and the increase of human demand for energy are becoming more and more serious, which force researcher to turn their attention to the reduction of CO_2_ and the development of renewable energy. Unsafety, easy to lead to secondary environmental pollution, cost inefficiency, and other problems limit the development of conventional CO_2_ capture technology. In recent years, many microorganisms have attracted much attention to capture CO_2_ and synthesize valuable products directly. Fatty acid derivatives (e.g., fatty acid esters, fatty alcohols, and aliphatic hydrocarbons), which can be used as a kind of environmentally friendly and renewable biofuels, are sustainable substitutes for fossil energy. In this review, conventional CO_2_ capture techniques pathways, microbial CO_2_ concentration mechanisms and fixation pathways were introduced. Then, the metabolic pathway and progress of direct production of fatty acid derivatives from CO_2_ in microbial cell factories were discussed. The synthetic biology means used to design engineering microorganisms and optimize their metabolic pathways were depicted, with final discussion on the potential of optoelectronic–microbial integrated capture and production systems.

## Introduction

More and more CO_2_ is emitted into the atmosphere due to rapid industrialization and high dependence on fossil fuels, resulting in many serious environmental problems (e.g., extreme weather patterns, greenhouse effect, sea level rise, ocean acidification, and ozone depletion) (Wu et al. 2020; Onyeaka and Ekwebelem 2022; Keasling et al. 2021; Pan et al. 2022). Moreover, the gradual depletion of fossil energy and a large amount of energy demand brought about by population growth is also a major challenge to human survival (Huang et al. 2022; Li et al. 2022a). Therefore, reducing CO_2_ in the air and finding alternative products of fossil energy has become urgent task for researchers.

Carbon capture technology can be used to capture CO_2_, thereby reducing CO_2_ in the air. In the industry, taking the power industry as an example, conventional carbon capture methods are usually divided into three categories according to the order of carbon capture and combustion process, including pre-combustion capture, oxygen-enriched combustion, and post-combustion capture (Thakur et al. 2018; Bhatia et al. 2019; Onyeaka and Ekwebelem 2022). The captured post-CO_2_ usually needs to be separated before it can be further used for storage or utilization. Conventional separation mainly includes physical methods (e.g., membrane separation, and low-temperature distillation) and chemical methods (e.g., absorption, and adsorption) (Bhatia et al. 2019). It should be noted that these conventional CO_2_ capture methods are unsafe and easy to give secondary environmental pollution, are cost-inefficient, and so on (Salehizadeh et al. 2020). The separated CO_2_ can be transported to a suitable location, such as the deep sea for storage. In addition to storing captured CO_2_, another more promising alternative to reducing carbon dioxide content in the air is to convert CO_2_ into fuels (Li et al. 2022b; Zhao et al. 2020; Sullivan et al. 2021; Hu et al. 2021). Among them, because of the excellent low-temperature startup, good lubricity, environmental protection, and safety, biodiesel is more and more recognized as an alternative to fossil energy (Tan et al. 2022; Abomohra et al. 2020). Biodiesel can be obtained from vegetable oil, animal fat, waste edible oil, and non-edible oil through a lipid exchange reaction (Wang et al. 2021a). Fatty acid esters (FAE), such as fatty methyl ester (FAME), fatty acid ethyl ester (FAEE), and fatty acid butyl ester (FABE), are considered to be one of the main components of biodiesel (Riaz et al. 2022; Sarwar et al. 2022; Zarska et al. 2022). In addition, fatty alcohols and aliphatic hydrocarbons are also considered to be sustainable biofuels (Krishnan et al. 2020; Liu and Li, 2020).

Plants and some microbes can mediate the conversion of CO_2_ into high-value products through their metabolic pathways, such as oil-producing yeast to furnish fatty acids (FAs) (Kim et al. 2022a). This provides a new idea of green, safe, reliable, and efficient production of fatty acid derivatives (Kim et al. 2019; Liu et al. 2021b). It is worth noting that due to a series of challenges such as the toxicity of industrial smoke and too high or too low CO_2_ concentration, the possibility of using microorganisms to achieve industrial direct production of biofuels is low. This requires reasonable modification of microorganisms to obtain efficient engineering microorganisms as microbial cell factories to capture CO_2_ and produce fatty acid derivatives. Of course, an accurate metabolic pathway, a clear enzyme mechanism, and an ideal microbial host are essential for the construction of a microbial cell factory (Liu et al. 2021c). Synthetic biology techniques such as CRISPR–Cas9 gene editing and high-throughput screening are considered to be one of efficient methods for constructing microbial cell factories and optimizing metabolic pathways (Chen et al. 2018; Ko et al. 2020).

**Fig. 1:**
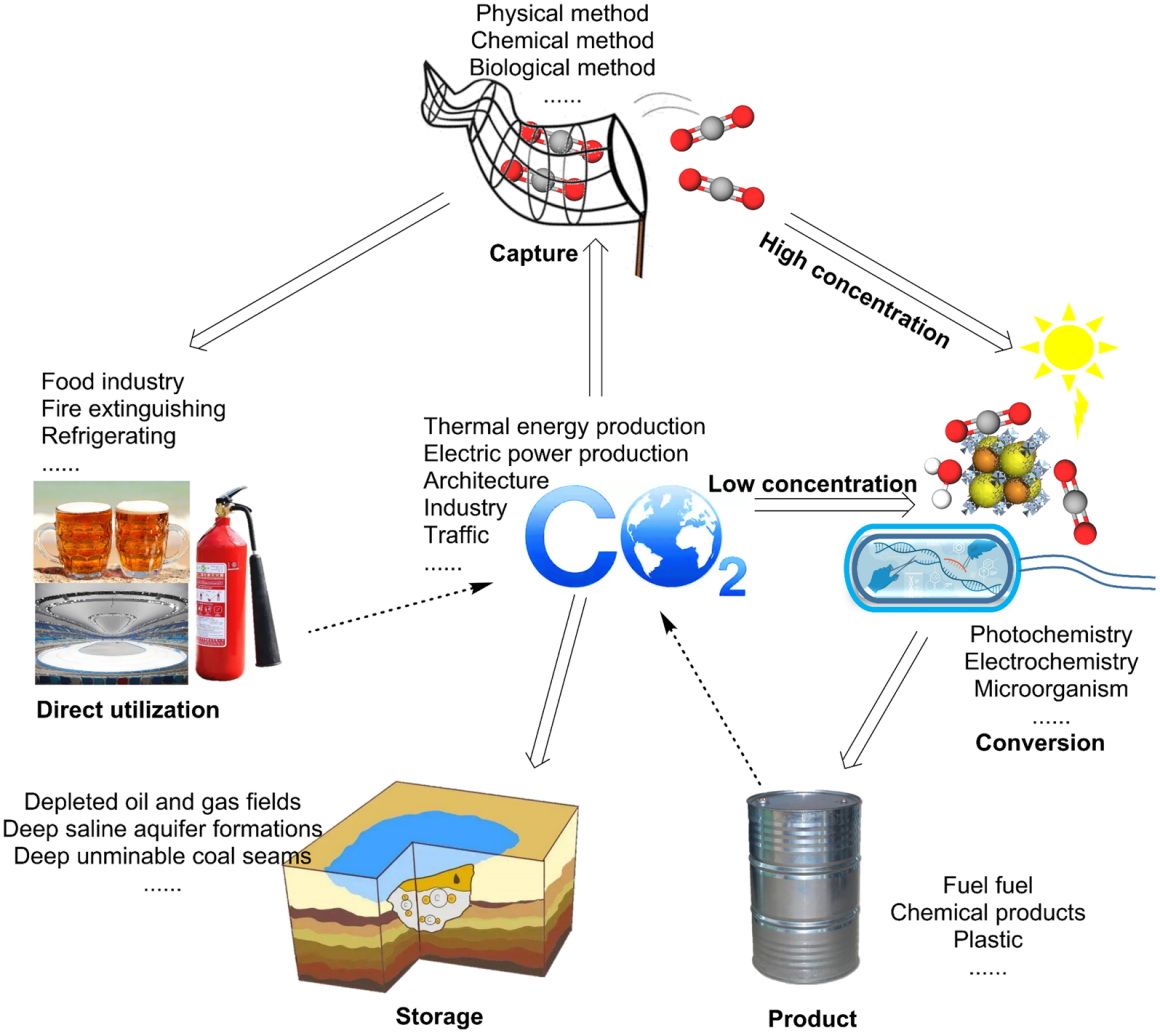
Conceptual map of capture, utilization and storage of CO_2_

In this review, we summarize the conventional CO_2_ capture and separation techniques, as well as the concentration mechanism and fixation pathways of CO_2_ in microorganisms. Then, the establishment of the pathway and progress for indirect and direct production of fatty acid derivatives in microbial cell factories are discussed. The tools and research progress of synthetic biology used to construct microbial cell factories and optimize metabolic pathways are also discussed in this review. Finally, the challenge of industrial production of fatty acid derivatives by engineering bacteria as a microbial cell factory through microbial transformation, and the possibility and the prospect of a hybrid system of optoelectronic–microbial integrated capture and production are discussed. The conceptual map of the capture, utilization, and storage of CO_2_ is shown in Fig. 1.

## Conventional capture technology and processing pathways

### Capture technology

Carbon capture mode and separation technology are important components of CO_2_ capture technology. As mentioned in the previous section, pre-combustion, oxygen-rich combustion, and post-combustion capture are the three main capture strategies in industrial processes reported today (Bhatia et al. 2019). Pre-combustion capture is the use of coal gasification and reforming reaction to separate and convert the carbon-containing components of the fuel into syngas dominated by H_2_, CO, and CO_2_ before combustion, and then the CO_2_ is separated using the appropriate separation technology while leaving H_2_ that can be used as a clean fuel (Giordano et al. 2019). High CO_2_ concentration, low separation difficulty, corresponding energy consumption, and cost reduction are the main advantages of this technology, but there is also the challenge of high investment cost, and the reliability needs to be improved (Roussanaly et al. 2019). Post-combustion capture usually refers to the direct separation of CO_2_ from the flue gas after combustion, which has less investment, but the low partial pressure of CO_2_ in flue gas leads to high energy consumption and high cost of carbon capture (Alalwan and Alminshid, 2021; Cheng et al. 2022). Oxygen-combustion assists flue gas circulation by separating oxygen from the air, and yields more concentrated CO_2_ (Alalwan and Alminshid 2021; Thakur et al. 2018). It can reduce the separation cost, but the total cost increases due to the need for independent air separation equipment to generate an oxygen-rich atmosphere (Alalwan and Alminshid 2021). Post-combustion capture technology is a widely used and mature technology at present, which only needs to add a CO_2_ trapping device after the existing combustion system without changing the original combustion mode, and has little change to the original system (Patrón and Ricardez-Sandoval 2022; Zhang et al. 2019).

The captured CO_2_ needs to be separated before utilization, and the traditional separation methods mainly include the physical and chemical methods (Alalwan and Alminshid 2021; Thakur et al. 2018). Among them, adsorption and absorption are the most widely used in industrialization (Vitillo et al. 2017; Zhang et al. 2020b). So far, the conventional adsorption methods mainly include physical and chemical methods. Physical absorption usually absorbs acidic gases under pressure to separate the carbon dioxide. For example, the solubility of CO_2_ in propylene carbonate can be absorbed or desorbed with pressure (Luo et al. 2021; Kolle et al. 2021). The principle of chemical adsorption to separate CO_2_ from flue gas is to use chemical reagents to react with CO_2_. Amino solvents (e.g., monoethanolamine, diethanolamine, methyl diethanolamine, and 2-amino-2-methyl-1-propanol) were once considered to be one of the most effective compounds for chemical absorption of CO_2_ in the thermal power industry (Daneshvar et al. 2022). The absorption method for the separation of CO_2_ has a good effect and high purity, but the high cost is a challenge in its industrial application (Shi et al. 2020). In addition, membrane separation technology is considered to be one of the most optimistic methods for carbon capture after combustion in coal-fired power plants because of its advantages, such as low energy consumption, low investment, and large contact area (Xu et al. 2019). The membrane separation method is mainly based on the selection of different components by the membrane material, and CO_2_ can pass through the membrane, and the remaining flue gas components cannot pass through the membrane (Thakur et al. 2018). Table 1 shows the foundational feature of different separation technologies or biological utilization.

**Table 1:** Foundational features of different methods for separation or utilization of CO_2_ (Bhatia et al. 2019; Thakur et al. 2018; Cheah et al. 2016)

Category	Method	Mechanism	Advantage	Insufficient
Physical method	Absorption	Henry's law. Usually, in absorbents, CO_2_ changes with pressure and temperature	Strong absorptive capacity, high selectivity, and simple operation	High energy consumption and cost
	Adsorption	Some solid adsorbents are selective to CO_2_ and can be desorbed with changes in temperature and pressure	Low energy consumption, simple operation, and controllable cost	Low selectivity and poor adsorption effect
	Membrane separation	The permeation rates of membrane materials to different gases are different	Simple operation, low energy consumption, and cost	Low durability of membrane materials
	Low-temperature distillation	The compressed and cooled CO_2_ is liquefied or solidified and then separated by distillation	Easy to operate and avoid the formation of by-products	High cost and low recovery rate of CO_2_
Chemical method	Absorption	CO_2_ can react with the absorbent and release CO_2_ again after heating	Strong absorption, good selectivity, mature and stable technology	Large loss of absorbent, high energy consumption, and cost
	Adsorption	Separation and recovery of CO_2_ components from a gas mixture by solid material adsorption or chemical reaction	Easy to operate and good adsorbability	Factors such as adsorption–desorption times and temperature have a too great influence on the performance
	Membrane absorption	Selective separation of CO_2_ by the combination of membrane contactor and chemical absorption	High selectivity, large contact area, and simple device	Low durability of membrane materials
	Electrochemical	CO_2_ was captured and separated by an electrochemical system	The technology is widely used and the separation cost is low	The electrode material is difficult to find and is highly corrosive at high temperatures
	Hydrate	Water and CO_2_ form CO_2_ hydrate at a certain temperature and pressure	A simple process has low energy consumption, good effect, and low loss of raw materials	The device is easy to corrode, and the requirement of material selection for equipment is high
Biological method	Enzyme catalysis	CO_2_ can be captured and transformed into substances such as formic acid by enzymes such as the RuBisCo enzyme and carbonic anhydrase	High efficiency, no by-products, strong specificity, the product can be used directly	The factors such as temperature have a great influence, the cost is high, and the controllability of the multi-enzyme system is low
	Whole-cell catalysis	Use cells (cyanobacteria, etc.) to capture and convert CO_2_ into products, such as biodiesel	Easy to operate, high efficiency, no by-products, strong specificity, the product can be used directly	The high temperature and toxicity of flue gas limit the growth of cells, and the transformation ability of natural cells is weak

### Processing pathways after capture

The means of carbon treatment in the process of industrialization are mainly related to carbon capture, utilization, and storage, that is, capture, transport, and storage (CCS), simultaneous utilization and storage after capture and transport (CCUS), and direct utilization after capture (CCU) (Daneshvar et al. 2022). The operation mode of CCS technology is to transport CO_2_ captured and separated from industrial and other point sources to storage sites for long-term storage to prevent CO_2_ from being emitted into the atmosphere (Daneshvar et al. 2022). Based on CCS, which also goes through the CO_2_ process of capturing and purifying emissions from the production process, CCUS has two more technical routes to put CO_2_ into the new production process to use or store (Nocito and Dibenedetto 2020). Deep-sea and underground geological storage are considered to be the best storage location (Daneshvar et al. 2022). In the current report, the common underground geological storage sites that seal the captured CO_2_ mainly include depleted oil and gas fields, deep salt aquifers, deep coal seams that cannot be exploited, and so on (Hoteit et al. 2019; Rathnaweera and Ranjith 2020; Mukherjee and Misra, 2018; Nocito and Dibenedetto 2020). Storing CO_2_ can effectively reduce the concentration of CO_2_ in the air, but many barriers urgently need to be broken, such as high cost and the risk of leakage, destruction, and alteration of geological layers (Daneshvar et al. 2022). CCU is different from CCS and CCUS in the final destination of CO_2_. In the CCU route, the CO_2_ captured from various industrial emission points will eventually be converted into valuable chemicals (FAEE, ethanol, methane, aliphatic alcohols, etc.) in theory (Zhang et al. 2020a). Chemical conversion, photocatalysis, electrocatalysis, photoelectrochemical and biological conversion are commonly used methods for the conversion of CO_2_ into valuable chemicals (Azhari et al. 2022; Wang et al. 2022; Das et al. 2020b; Kumaravel et al. 2020; Bhatia et al. 2019).

## Concentration mechanism and fixed pathway of CO_2_ by microorganisms

### CO_2_ concentration mechanism

**Fig. 2:**
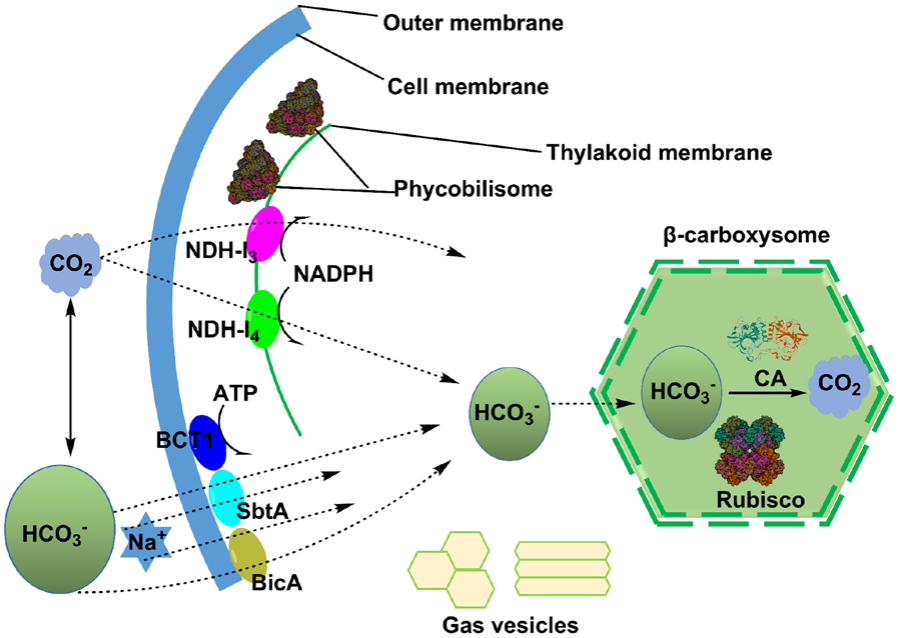
CO_2_ concentration mechanism of a *cyanobacteria*

TO cope with adverse factors, such as environmental stress (e.g., low CO_2_ concentration) and low CO_2_ affinity of microorganisms, some autotrophic microorganisms (e.g., cyanobacteria) have evolved a CO_2_ concentration mechanism to ensure the CO_2_ of carbon fixation pathway. To date, a variety of CO_2_ concentration mechanisms have been reported, usually achieved by the use of Rubisco enzymes (ribulose bisphosphate carboxylase/oxygenase) to gather CO_2_ around it, which can be divided into direct CO_2_ absorption and absorption of bicarbonate (Burlacot et al. 2022; Wu, 2021; Wei et al. 2019; Liu 2022). Taking cyanobacteria as an example, there are five CO_2_ pathways of cyanobacteria, three of which are concentrated by bicarbonate and two of which absorb CO_2_ are directly shown in Fig. 2 (Huisman et al. 2018).

### CO_2_ fixed pathways

**Fig. 3:**
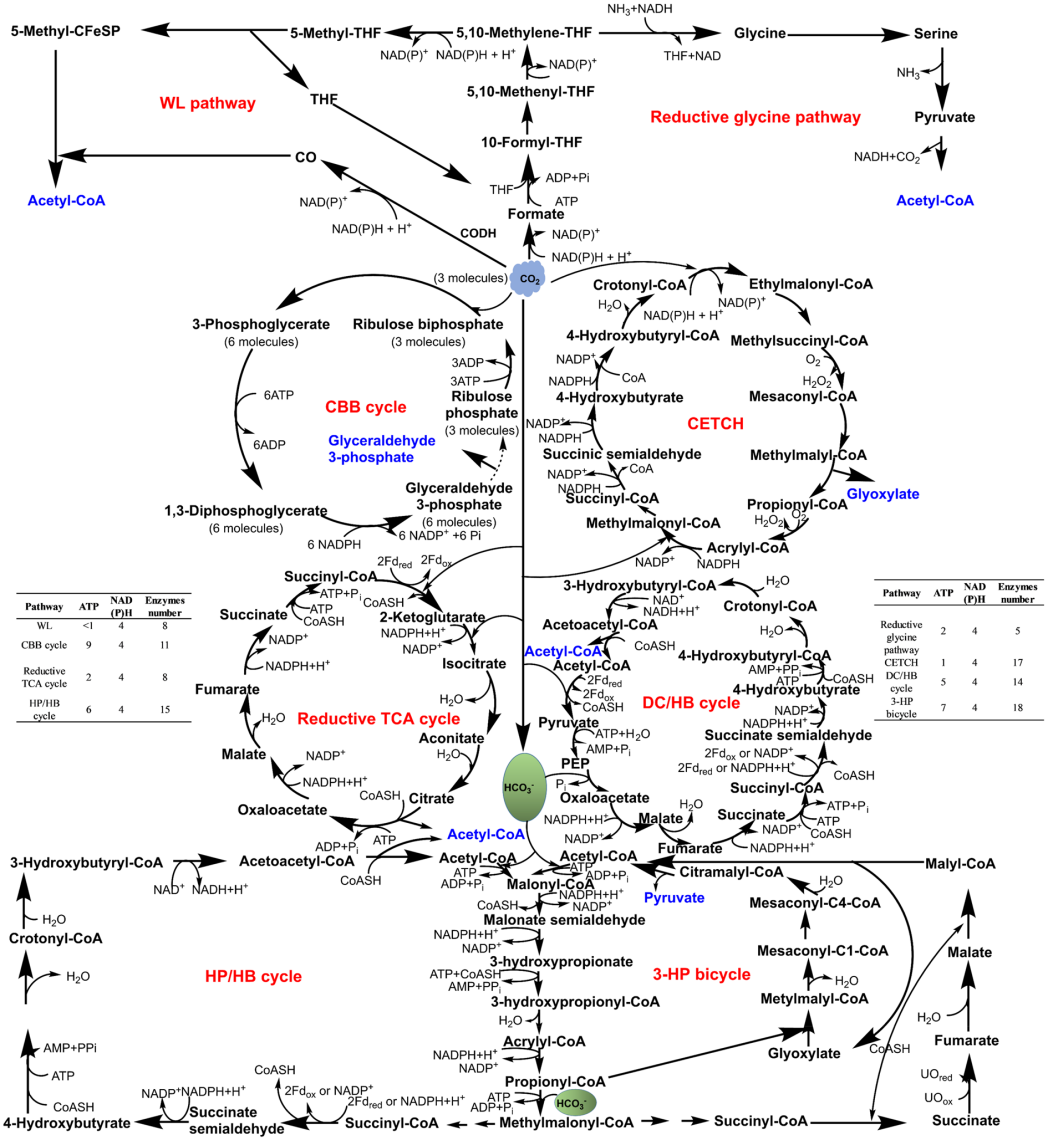
Common fixed pathway of CO_2_. The number of enzymes, the amount of ATP consumed and the reduction equivalent are given, and the metabolic spillover products of each pathway are represented in blue font

As shown in Fig. 3, the commonly used CO_2_ fixed paths reported so far are usually divided into six categories, in accordance with their characteristics, such as topology, carbon fixation pathway, and fixed carbon species, namely, CBB (Calvin–Benson–Bassham) cycle, HP/HB (3-hydroxypropionic acid/4-hydroxybutyrate) and 3-HP (3-hydroxypropionic acid) cycle, rTCA (reduced tricarboxylic acid) cycle, WL (Wood–LJungdahl) pathway and reduced glycine pathway (rGly), DC/HB (dicarboxylate/4-hydroxybutyrate) cycle, and CETCH (crotonyl-CoA/ethylmalonyl-CoA/hydroxybutyryl-CoA) cycle (Liu et al. 2020; Salehizadeh et al. 2020). CBB is the most common CO_2_ fixed pathway, which captures CO_2_ as a carbon source and produces sugars, which are widely found in green plants, cyanobacteria, algae, purple bacteria, and some amoeba (Salehizadeh et al. 2020). It mainly uses sunlight as an energy source, and rubisco and ribulose phosphatases play a key role in this process (Liu et al. 2020; Antonovsky et al. 2016; Yu et al. 2020). The WL pathway is similar to the reductive glycine pathway in topology, and they are rare anaerobic fixed pathways for the direct reduction of CO_2_ (Chen et al. 2021; Smith et al. 2019; Claassens, 2021). For example, CO_2_ can be reduced and attached to a C1 carrier, and then C2 compounds can be obtained by connecting to another CO_2_ molecule. The key enzymes in the WL pathway mainly include CO dehydrogenase, formylmethane furan dehydrogenase, formate dehydrogenase, and so on (Jiao et al. 2021), while glycine synthase is the key enzyme in the rGly pathway (Sanchez-Andrea et al. 2020). A common feature of DC/4-HB, 3HP-4-HB, 3-HP/malyl-CoA cycle, and rTCA pathway is that their evolution revolves around a common intermediate, and it can be determined that the two conservative metabolites succinyl-CoA and acetyl-CoA are very important in these pathways, and the sharing of several reactions between each cycle and another cycle is another common characteristic in this group (Liu et al. 2020). CETCH is a classical synthetic pathway of fixed CO_2_ verified in vitro, and 17 enzymes are recombined in this pathway, of which crotonyl-CoA carboxylase/reductase is one of the key enzymes (Liu et al. 2020; Schwander et al. 2016). What needs to be paid attention to is that three pathways (3HP–4HB, CBB, and 3-HP/malyl-CoA) are aerobic, another three pathways (WL, rTCA, and DC/4H) are anaerobic, and the last two pathways (CETCH, and rGly) are synthetic (Salehizadeh et al. 2020). Of course, in addition to the above carbon fixation pathways, other clear or unidentified fixed pathways have been reported, especially with advances in genomes, biochemical models, and other synthetic biology tools, CO_2_ fixation pathways may need to be re-evaluated (Liu et al. 2020; Jatain et al. 2021).

## Conversion of CO_2_ to fatty acid derivatives by microorganisms

### Indirect production by microorganisms

Microbial oil is extracted from fast-growing microorganisms and can be transesterified with alcohols (e.g., methanol, ethanol, and butanol) to produce FAE (Ma et al. 2018). Similarly, microbial oils can be converted into fatty alcohols and aliphatic hydrocarbons by chemical reactions, such as catalytic hydrogenation and catalytic decarboxylation (Chen et al. 2022a; Munkajohnpong et al. 2020; Liu and Li 2020). So far, many oil-producing microorganisms, including bacteria, microalgae, yeast, and filamentous fungi, have been widely reported, and they can accumulate intracellular fat that accounts for a large part of their biomass (Manish et al. 2018).

#### Microalgae

Microalgae are usually defined as unicellular or multicellular microorganisms (Kim et al. 2022b; Hu et al. 2018; Koreti et al. 2022), although other studies define them as primitive plants (Zheng et al. 2022). Microalgae is a kind of photoautotrophic microorganism, which can convert CO_2_ into bio-oils, mainly including *prokaryotic cyanobacteria*, *green algae* (*cyanobacteria*), *eukaryotic microalgae*, etc., and are considered by many researchers to have great potential in the production of biofuels (Wijffels and Barbosa 2010; Nitsos et al. 2020). Compared with plants, the competence of CO_2_ fixation by microalgae is higher, and the land area needed for planting is smaller. Compared with macroalgae, microalgae have higher oil content and a faster growth rate. For example, compared with sesame seeds (50.1–52.3%), safflower oil (35.4–39.5%), sunflower oil (24.0–46.8%), and other plants, the oil content of microalgae is usually higher, generally, between 20–70% of dry biomass, and even some microalgae contain more than 80% of dry weight (Ravanipour et al. 2021; Pal et al. 2019; Taparia et al. 2016). Of course, different species of microalgae obtain different lipids, compound oils, and hydrocarbons. For example, Anahas and Muralitharan (2018) evaluated the potential of 15 strains of heterocrystalline cyanobacteria in FAEE and hydrocarbon production and measured FA (fatty acid) composition, FAEE quality, and biomass, fat, and hydrocarbon production. The results showed that SA (stearic acid, 14.64–69.84%) and PA (palmitic acid, 3.79–40.84%) were the most favorable substances for FAEE production among the tested cyanobacteria. Rodríguez-Palacio et al. (2022) evaluated their ability to produce various types of biomass, especially lipids, by culturing *Desmodesmus quadricauda, Chlorella*, *Coelastrella sp.*, *Neochloris oleoabundans,* and *Verrucodesmus verrucosus*. The results showed that the total fat of *Coelastrella sp* was composed of 82.9% C_16_–C_18_ carbon chain FAs, and the proportion of C18V3 was less than 12%. However, in the results of *Neochloris oleoabundans*, an increase in FAs with unsaturated chain (C18:2) was shown, which exceeded the 12% limit in the case of C18:3. Most microalgae use the CBB pathway to fix CO_2_, which is a process of using ATP and NADPH generated by light to synthesize organic compounds. In this way, CO_2_ can be fixed and glyceraldehyde3-phosphate can be obtained, while free fatty acids can be obtained further (Liu et al. 2020). Microalgae usually change their lipid biosynthesis pathway under adverse or stress conditions to form and accumulate neutral lipids (20% of stem cell mass). This neutral lipid is usually converted into TAG (triglyceride) in cells as a form of carbon and energy storage. The three independent regulatory steps of FAs biosynthesis, glycerol formation, and packaging into lipid droplets (LDs) are usually the TAG biosynthesis process (Chen and Wang 2021). In addition, with the extensive application of biotechnology, such as the means of synthetic biology, metabolic engineering and enzyme engineering in the oil production of microalgae, there is great potential for the production of FAEE through microalgae.

#### Bacteria

More and more kinds of oil-producing bacteria have been confirmed, which can be used as precursors of FAs, and then further synthesize fatty acid derivatives by lipid exchange reaction, catalysis, and other reactions. Of course, many bacteria produce complex lipids, which is a big challenge to be directly used in fatty acid derivatives production, and a large number of these bacteria are heterotrophic bacteria that cannot directly convert CO_2_ into bio-oils (Koreti et al. 2022). Among the autotrophic microorganisms that can fix CO_2_, the CBB, the rTCA, the WL, and the 3-HP/malyl-CoA cycle are four common pathways of carbon sequestration (Manish et al. 2018). The CBB pathway is usually used to fix carbon by purple non-sulfur bacteria (*Rhodobacter*, *Rhodopseudomonas*) and Thiobacillus purpura (*chromatin bacilli*), as well as hydrogen bacteria (*Rosella*, *hydrogen gene vibrio*) and other chemical autotrophic bacteria (such as *Thiobacillus*). Green sulfur bacteria and hydrophilic bacteria usually use the rTCA pathway to fix carbon, while the WL pathway has proven to be found in many bacteria, such as *Proteus*, *Clostridium thermophilus,* and so on. The 3-HP/malyl-CoA cycle is mainly found in some green non-sulfur bacteria of the family *Chloroflexaceae*. Some bacteria (e.g., *Bacillus SS105*, *Pseudomonas sp*, and *Serratia ISTD04*) can convert fixed CO_2_ into microbial oil by metabolism, and then obtain FAEE by lipid exchange reaction (Manish et al. 2018; Salehizadeh et al. 2020). Maheshwari et al. (2018) isolated the bacterial strain *Bacillus sp.* from free-air CO_2_-concentrated soil and screened it for carbon dioxide sequestration and FAs production. The results showed that the *Bacillus SS105* strain exhibited higher lipid production ability, with a total fatty acid content of 120 mg/L, saturated fatty acid content of 51%, unsaturated fatty acid content of 49%, and a high proportion of PA (37.80%) and OA (oleic acid, 37.88%). Kumar et al. (2017) obtained a strain of *Serratiasp. ISTD04 is* capable of transforming CO_2_ into FAEE from the bacterial community of Paleoproterozoic metamorphic rocks. GC–MS analysis indicated that C13–C24 and C11–C19 were in the main range of hydrocarbons and FAMEs produced in bacteria, respectively. 59% are saturated organic compounds and 41% are unsaturated organic compounds.

In addition to microalgae and bacteria, many fungi have also been reported to have the ability to produce oil, such as oil-producing yeast, Aspergillus oryzae, and so on (Abeln and Chuck, 2021; Malik et al. 2022). Unfortunately, most of the reported fungi are heterotrophic microorganisms and usually do not have the ability to assimilate CO_2_. Of course, the engineering yeast modified by biotechnology no longer has such a problem (Gassler et al. 2020; Xie 2017).

### Direct production in microbial cell factory

The direct production of fatty acid derivatives by microorganisms is accomplished by introducing the production pathway or key enzyme genes of FAE, alcohols, fatty alcohols, and aliphatic hydrocarbons into the host (e.g., microalgae, and purple non-sulfur bacteria). Taking the production of FAEE as an example, the production of FAEE through microbial cell factories is mainly through the introduction of synthetic metabolic pathways employing biotechnology (e.g., synthetic biology, and metabolic engineering) to complete the production of endogenous ethanol and fatty acids in the cell factory. And then, FAEE is further synthesized in the presence of the enzyme (usually wax ester synthase/acyl coenzyme A, abbreviated as WS/DGAT). Table 2 shows the basic situation of some microorganisms using CO_2_ to produce biofuels.

**Table 2:** Production of biofuels by some microorganisms using CO_2_

Host	Product	Titer (g/L)	Yield (%)	Productivity (mg/L/d)	Cultivation condition			References
					Medium	Light (μmol/m^2^/s)	Temperature (℃)	
*D. quadricauda*	Lipids	3.5			BF	50	36	Rodríguez-Palacio et al. (2022)
*Bacillus sp.* SS105	Lipids	0.38^a^	38.8	59.29	MSM	100	30	Maheshwari et al. (2018)
*Serratia sp.* ISTD04	Lipids	0.72^a^			LB		30	Kumar et al. (2017)
*Synechocystis sp.* PCC6803	Alcohol	5.50		212	BG-11		37	Gao et al. (2012)
*S. elongatus*	1-butanol	0.71			BG-11		30	Lan and Liao (2012)
*R. eutropha* H16	Isobutanol and 3-methyl-1-butanol	1.4			German minimal		30	Li et al. (2012)
*Synechococcus elongatus*	Isobutanol	0.64^a^			BG-11	50	37	Wu et al. (2021)
*Rhodopseudomonas palustris* TIE-1	n-butanol	0.14^a^	4.9	12^a^	YP		30	Bai et al. (2021)
*E. coli*	Lipids	0.5			M9		70	Lee et al. (2020)
*Synechococcus elongatus* PCC 7942	FAEE	0.017			BG-11	100	30	Lee et al. (2017)
*Synechocystis sp.* PCC 6803	FAME	0.12	67		BG-11	60	30	Yunus et al. (2020)
*Synechocystis sp.* PCC6803	FAME	4.05			BG-11	35	30	Kang et al. (2021)

^a^The value is calculated based on the reported data

#### Synthesis of FAs by the microorganism

**Fig. 4:**
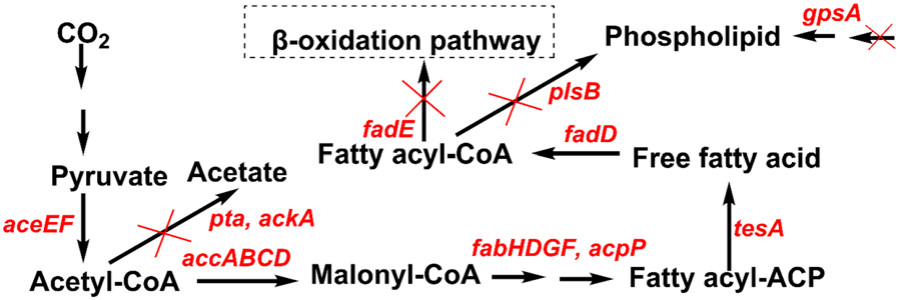
Schematic diagram of the construction of a microbial metabolic pathway for the production of fatty acids. Two arrows represent multiple steps

As in the previous section, some microalgae, bacteria, and fungi can synthesize bio-oils through their metabolic pathways. Most FAs produced in microbial cells are commonly used to synthesize phospholipids and TAGs, and of course, some are degraded through the beta-oxidation pathway. Therefore, it is necessary to obtain efficient production of FAs by biotechnology (e.g., genetic engineering, metabolic engineering, and enzyme engineering). Introducing or modifying the genes of major enzymes in the pathway of fatty acid synthesis is an effective means to achieving efficient production of FAs by engineering bacteria (Santos-Merino et al. 2018). The synthesis of FAs by microorganisms involves genes of a variety of enzymes, and among them, *fadE* encodes the *β*-oxidation pathway, *tesA* encodes thioesterase, *fadD* encodes acyl-CoA synthetase, *accABCD* is used for acetyl-CoA carboxylase, *fabH*, *fabD*, *fabG,* and *fabF* are genes of fatty acid synthetase, and *aceEF* is related to the coding of pyruvate dehydrogenase and pyruvate formate lyase, and the *gpsA*, *plsB*, *ptA,* and *ackA* are used to encode glycerol-3-phosphate dehydrogenase, glycerol-3-phosphate *O*-acyltransferase, phosphate transferase, and acetate kinase, respectively (Intasian et al. 2021; Gajewski et al. 2017; Zhuang et al. 2022b, 2022c). Acetyl-CoA is directly involved in the synthesis of FAs, and fatty acid synthase is the key enzyme in the synthesis of FAs. FAs synthase is a multifunctional enzyme with seven different catalytic domains and one acyl carrier protein domain with a molecular weight of about 2.7 million Dalton and can be assembled into a cage supramolecular structure. Figure 4 shows the construction of metabolic pathways for FAs production by engineering microorganisms.

#### Synthesis of fatty alcohols and aliphatic hydrocarbons by the microorganism

**Fig. 5:**
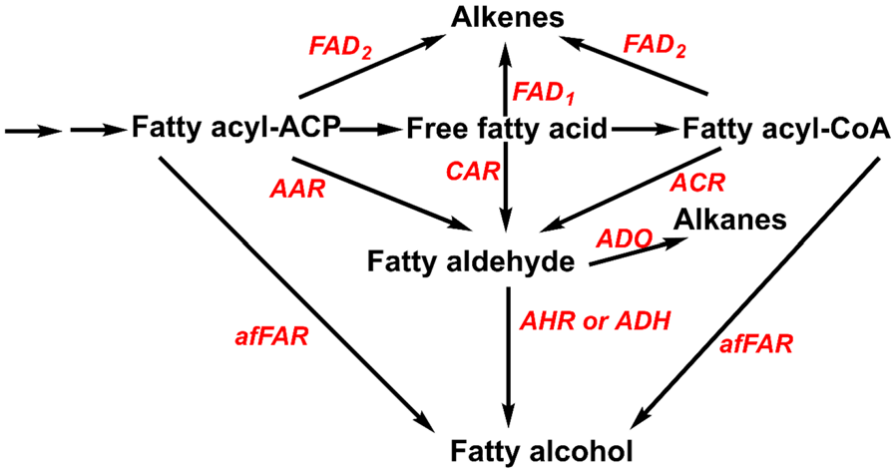
Schematic diagram of the synthetic pathway of fatty alcohols and aliphatic hydrocarbons production by microorganisms. Two arrows represent multiple steps

Microorganisms mainly produce fatty alcohols through fermentation. *Saccharomyces*, *Schizosaccharomyces*, *Candida,* and *Aspergillus* other microorganisms are common microorganisms used for fermentation to produce fatty alcohols. In addition, *Clostridium thermocellum* and *Zymomonas mobilis* are also reported to be used in the synthesis of fatty alcohols (Hon et al. 2017; Szambelan et al. 2022). Carbohydrates (e.g., glucose, and xylose) are usually the main carbon sources for fatty alcohol synthesis by microbial fermentation. Taking ethanol production as an example, glucose is converted to pyruvate through microbial metabolic pathways (e.g., glycolysis, and Entner–Doudoroff pathway). Acetaldehyde is obtained after further decarboxylation of pyruvate, which is then finally reduced to ethanol (Valk et al. 2022; Yan et al. 2022). Unfortunately, these natural microbial metabolic pathways are usually unable to directly use CO_2_ as a carbon source to synthesize the target product. Optimizing metabolic pathways by biotechnology can solve this problem very well, AAR/ACR (acyl-ACP/CoA reductase), CAR (carboxylic acid reductase), AHR (aldehyde reductase), ADH (alcohol dehydrogenase) and afFAR (alcohol-forming fatty acyl-CoA reductase) are the key enzymes that need to be optimized or introduced in the design of this pathway shown in Fig. 5. For example, Gao et al. (2012) used an important *cyanobacterial* model (*Synechococcus PCC6803*) as a parent algal strain. The *PdcZM–AdhIIZM* (pyruvate decarboxylase-type II alcohol dehydrogenase) pathway from *Zymomonas mobilis* was introduced into *Synechocystis sp.* PCC6803 and the pathway for ethanol synthesis by photosynthesis was successfully obtained. Luan et al. (2015) characterized and optimized the *PDChim–slr1192* pathway which consists of ketoacid decarboxylase (*PDChim*) and ethanol dehydrogenase II (*Slr1192*) in engineering cyanobacteria. The results show that to further improve its ethanol synthesis ability, it is necessary to further improve its activity, increase its proportion and keep it at about 1:1.5. The microbial synthesis of higher alcohols (C_3+_ fatty alcohols) is similar to the synthesis of ethanol, which is also achieved by introducing or optimizing the metabolic pathway of higher alcohols in the host. Lan and Liao (2012) promoted the reversal of *β*-oxidation by modifying the ATP consumption to acquire 1-butanol under the photosynthetic conditions using *cyanobacteria* PCC 7942. Li et al. (2012) modified *Ralstonia eutropha* H16 by genetic engineering technology to produce isobutanol and isopentanol. Wu et al. (2021) constructed an engineering strain of *Chlorella elongate,* using CO_2_ as the carbon source to synthesize isobutanol, and found that 2% sea salt stress could significantly increase isobutanol production by 5 times. Bai et al. (2021) introduced the gene of the *n*-butanol production pathway into *Rhodopseudomonas palustris* TIE-1 (photoautotrophic organism) to realize the production of *n*-butanol. As for the biosynthesis of methanol by CO_2_, its production in microbial cell factories is limited due to toxic, hazardous, and other factors, so there are no reports on the direct use of CO_2_ to synthesize methanol (Jiang et al. 2021). Of course, with the rapid development of bioengineering and technology (e.g., synthetic biology, and enzyme engineering), it is possible to produce FAME by engineering microorganisms with artificial methanol pathways in the future. In addition, some studies have shown that the method of bioconversion of methanol to FAs derivatives has been obtained (Gao et al. 2022a; Cai et al. 2022).

Similar to the biosynthesis of fatty alcohols, the biosynthesis pathway of aliphatic hydrocarbons is to introduce or optimize the enzymatic reaction pathway of FAs (e.g., decarboxylation, and oxygenation) based on the FAs synthesis pathway. ADO (aldehyde deformylating oxygenase) and FAD (fatty acid decarboxylaseare) is the key enzyme in this process. Zhou et al. (2016) convert fatty acids to aliphatic aldehydes by building a yeast cell factory, which is then further converted into aliphatic hydrocarbons (0.8 mg/L titer) and fatty alcohols (1.5 g/L titer). Gao et al. (2020) believed that the production of aliphatic hydrocarbons was first catalyzed by AAR to reduce fatty acyl-ACP protein or fatty acyl-CoA to long-chain aliphatic aldehydes, and then to aliphatic hydrocarbons catalyzed by ADO.

#### Synthesis of FAE by the microorganism

**Fig. 6:**
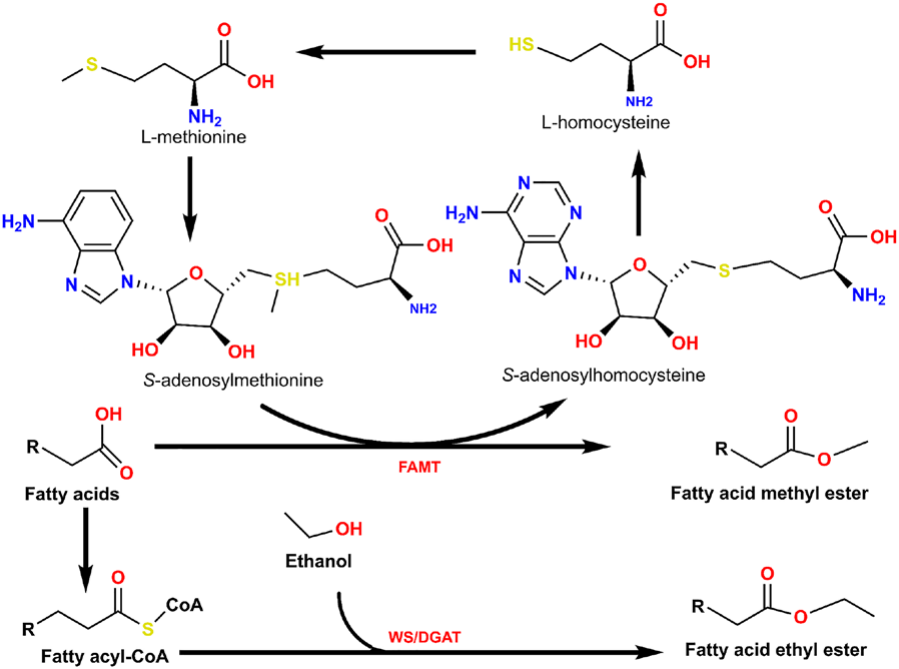
Schematic diagram of the production of FAME and FAEE by an engineering microorganism

Most higher alcohols are usually directly used as biofuels or fuel additives owing to their properties (e.g., high energy density, non-hygroscopicity, and low corrosiveness) (Zhao et al. 2022; Liang et al. 2020b; Gonçalves and Simões, 2017; Kumar and Saravanan, 2016). Therefore, this section focuses on the construction or optimization of biodiesel production pathways, such as FAME and FAEE in microbial cell factories (Fig. 6).

Unlike the conventional synthesis mode of FAEE by transesterification of oils produced by plants, animals, or microorganisms with ethanol under the action of appropriate catalysts, the production of FAEE by engineering microorganisms is usually to introduce or optimize the metabolic pathway that can produce ethanol and FAs at the same time and introduce *atfA* gene to synthesize WS/DGAT (multifunctional wax ester synthase/acyl coenzyme A: diacylglycerol acetyltransferase) and further synthesize FAEE in cells (Wang et al. 2020a). In general, the development of engineering strains for the production of FAEE requires two main steps (Kim et al. 2019). The first step is that FAs are activated to fatty acyl-CoA, which is usually accomplished by fatty acyl-CoA synthetase encoded by *fadD* gene, while preventing fatty acyl-CoA dehydrogenase encoded by *fadE* gene from degrading fatty acyl-CoA. The second step is to use WS/DGAT to further condensation of the formed fatty acyl-COAS with ethanol to obtain FAEE. Different from the ethanol pathway required for the conversion of FAs to FAEE, the production of FAME requires the conversion of FAs with *S*-Adenosylmethionine as a methyl donor under the action of FAMT (fatty acid methyltransferase). Some ideal bacteria and fungi (yeast, *E. coli*, etc.) have been reported as host strains to construct microbial cell factories to produce FAE. For example, Kadisch et al. (2017) knocked out the hydrolase BioH in *E. coli* to achieve effective FAME bioprocessing. Rahman et al. (2019) conducted experiments to explore how the biosynthesis of FAEE in *E. coli* is affected by engineering fatty acid operons (*fabHDG*). The experimental results show that the strains with highly expressed operons composed of *fabH*, *fabD*, and *fabG* genes showed the best FAEE synthesis ability, with a yield of 1291 mg/L. Zhang et al. (2021) successfully expressed the *WS/DGAT* gene of *Acinetobacter baylyi* by introducing various wax ester synthase genes from different sources to construct the synthetic pathway of FAEE in *Rhodotorula toruloides*. The results showed that the final titer of the highest yield of FAEE was 9.97 g/L, and most of the FAEE was secreted from extracellular cells. In addition to yeast and *E. coli*, Xu et al. (2021) obtained a strain of Aspergillus Niger that could produce FAEE through screening experiments.

In most of the reported host microbes (e.g., *E. coli*, and yeast) used to build FAE microbial cell factories, organic compounds (glucose, etc.) are still the mainstream carbon sources. Of course, it has also been reported that engineering strains that is able to convert CO_2_ into target chemicals can be constructed by introducing CO_2_ fixed pathway into these microorganisms. For example, Lee et al. (2020) introduced the synthetic fixed CO_2_ pathway (the CBB pathway of *Rhodobacter sphaeroides*) into the industrial platform microbial *E. coli* to obtain biofuel production through photosynthesis. Besides, because the construction procedure of engineering strain for producing FAE is relatively simple and effective, autotrophic microorganisms (*Chlorella*, *cyanobacteria*, etc.) attracted the attention of researchers (Joshi and Mishra, 2022). Compared with the mode that heterotrophic engineering microorganisms convert organic matter into intermediates (e.g., pyruvate) through metabolic pathways (e.g., glycolysis pathway) and then further into FAE, autotrophic engineering strains obtain intermediates through CO_2_ immobilization pathways (e.g., the CBB, and 3-HP/malyl-CoA cycle pathways) and further synthesize FAEE (Meng et al. 2022; Peng et al. 2019; Nisar et al. 2021; Joshi and Mishra, 2022). Lee et al. (2017) modified *Synechococcus elongatus* PCC 7942 by expressing iso-wax ester synthase and introducing ethanol pathway to obtain an engineering cyanobacteria strain for producing FAEE. Yunus et al. (2020) constructed an engineering cyanobacteria using CO_2_ to produce FAME. The production process was carried out using ADOMet as a methyl donor under the action of FAMT, and the results showed that 120 mg/L of FAME could be produced within 10 days. Similarly, Kang et al. (2021) constructed engineering cyanobacteria by introducing the FAMT gene and converting FAs into FAME in vivo. In addition to photoautotrophic microorganisms, such as microalgae, it has also been reported that chemical autotrophic and heterotrophic microorganisms have been transformed into engineering bacteria that can produce FAE, such as *Cupriavidus necator* H16 (Panich et al. 2021; Brigham 2019).

## Synthetic biology tools for constructing and optimizing microbial cell factories

### Construction technology of microbial cell factory

Synthetic biology tools such as genome editing technology, multi-gene simultaneous regulation technology, protein skeleton technology, and high-throughput screening technology are commonly reported as microbial cell factory technologies (Liu et al. 2021a; Jung et al. 2021; Su et al. 2017).

#### Genome editing technology

The commonly used genome editing techniques include homologous recombination technology, zinc finger nuclease technology, transcriptional activator effect nuclease technology, and so on (Jeon et al. 2017; Gao et al. 2022b; Paschon et al. 2019; Liu et al. 2017). However, the time consumption, high cost, and host limitations of these technologies limit the efficient construction of microbial cell factories. Therefore, CRISPR gene editing technology has been deeply developed and widely used in recent years due to its simple operation, high efficiency of gene editing, low cost, and so on (Koch 2021; Wang et al. 2021b; Liu et al. 2021c). For instance, genomic editing of *Halomonas spp.* by CRISPR/Cas9 system can efficiently produce microbial copolymer P (3HB-co-3HV, consisting of 3-hydroxybutyrate and 3-hydroxyvalerate) (Qin et al. 2018).

#### Simultaneous regulation of multiple genes

It is usually difficult for a single enzyme to complete the biosynthesis of FAEE, and its biosynthetic pathway usually depends on the coordination of multiple enzymes to balance. Therefore, the overexpression of a single gene through plasmid can easily lead to an excessive metabolic load of cells, which is not conducive to growth metabolism and product synthesis. The simultaneous regulation of multiple genes can well solve this problem and reasonably regulate the expression balance of metabolic pathways (Jung et al. 2021). For example, Warner et al. developed a genome-traceable multivariate recombination engineering technique that can analyze and modify thousands of sites at the same time (Warner et al. 2010).

#### Protein skeleton technology

In the microbial cell factory, the distance between the enzymes involved in the reaction and the substrate and the spatial position of the adjacent enzymes in the synthetic pathway usually affect the efficiency of the metabolic pathway to a great extent (Liu et al. 2022a). The enzyme is anchored to the synthetic protein skeleton according to a specific spatial position, to make the related enzymes gather in a specific area, then increase the probability of binding between the enzyme and the substrate, and greatly improve the synthesis rate of the product. At the same time, the protein skeleton can also regulate the catalytic efficiency of the enzyme, obtain the optimal combination of catalytic efficiency, and finally improve the synthesis efficiency of the product. In the study of Lee et al. (2018), a supramolecular scaffold system was infiltrated into the entire *E. coli* cytoplasm, increasing the possibility of enzyme binding to the substrate. Kang et al. (2019) constructed a multienzyme complex by selecting a pair of short peptide tags (RIAD and RIDD) to increase carotenoid production and control the flow of metabolites.

#### Gene dynamic regulation technology

To enable cells to timely and more accurately perceive the changes in external environmental conditions, and turn on or off gene expression at an appropriate time, to realize the dynamic regulation of metabolic pathways, one of the basic ideas provided by this regulation technology is to design an artificial gene circuit (Yeom et al. 2021). The basic idea of this regulation technology is to design an artificial gene circuit, enable cells to sense changes in external environmental conditions and turn on or off gene expression at an appropriate time to realize the dynamic regulation of metabolic pathways (Jung et al. 2021). For example, Zhang et al. (2012) constructed an engineering *Escherichia coli* to produce an FAs-based product, and this construction process was based on the acquisition of a dynamic sensor regulation system (DSR).

#### The high throughput screening technique

High-throughput screening technology is considered an effective auxiliary means for the rapid construction of microbial cell factories, which can quickly and efficiently screen out microbial strains that are more suitable for industrial production. Based on a molecular level and cellular level, this technology takes the form of a microplate as the carrier of experimental tools, through the automatic operating system, which uses sensitive and rapid detection instruments to collect data and analyze and process them by computer (Sarnaik et al. 2020). Using this technology, a large number of data can be detected at the same time, and it has the characteristics of tracking, fast, sensitive, and accurate (Zeng et al. 2020). Schmidl et al. (2019) used modular DNA combined with domain exchange to reconnect bacterial two-component systems for activity detection of gene routes, while Kan et al. (2016) applied it to the activity detection of the original enzyme.

### Optimization of the production path

In microbial cell factories, the metabolic pathway for the synthesis of fatty acid derivatives is usually reconstructed by heteroenzymes, which usually results in the accumulation of intermediates, growth retardation and metabolic imbalance. The tools of synthetic biology are becoming more and more mature and widely used, which opens up a new scheme for engineering microorganisms to obtain the systematic optimization of the metabolic pathway, thus realizing the balance between cell networks and chemical biosynthesis (Ko et al. 2020). According to genetic hierarchical control, these tools can be roughly divided into six types of engineering (DNA level, RNA level, protein level, metabolite level, genome level, and cellular level) (Chen et al. 2018). The engineering at the DNA level mainly includes promoter engineering. By starting the project to optimize the metabolic pathway, the potential of microbial plants to produce biofuels, especially FAEE, has been further enhanced (Srivastava et al. 2020; Quinn et al. 2019; Shen et al. 2018). Construction of promoter libraries with different intensities, promoter substitution, synthesis of RBS (ribosome binding site on mRNA) regulation, and gene spacer regions are common strategies in promoter engineering (Chen et al. 2018). Transcription factor engineering and RNA switch synthesis are the main initiation projects at the RNA level. Transcription speed is usually regulated by the interaction between transcription factors and the promoter region of target genes, while transcription factor sequence-specific proteins are usually composed of a DNA-binding domain, transcriptional regulatory domain, and nuclear localization signals (Chen et al. 2018; Deng et al. 2022). Reasonable regulation of various enzymes in metabolic pathways and transcription factors can promote the production of biofuels, such as FAEE in microbial cell factories (Mochdia and Tamaki 2021; Li et al. 2019). Generally speaking, zinc finger protein transcription factor, *myb* and *bHLH* transcription factor, and *ORCA* protein are the key technical nodes of the transcription factor engineering strategy. It is worth noting that *myb*, *bHLH*, and *ORCA* proteins are mainly used to regulate plant growth and development, physiological metabolism, cell morphology, and model formation (Chen et al. 2018; Wang et al. 2020b). Zinc finger protein transcription factors are usually composed of highly conserved carboxyl terminals and different amino terminals containing 4–6 zinc fingers. This structure facilitates interaction or binding with DNA, RNA, or other proteins (Feldman et al. 2019). Another project at the RNA level is the synthesis of RNA switches. Small molecule RNA, ribose switches, RNA interference, and antisense RNA techniques are usually the main regulation strategies of this tool (Chen et al. 2018).

The optimization technology at the protein level is mainly to change the properties of proteins by customizing protein "components" and protein "devices" to meet the needs of synthetic metabolic pathways, and through this technology, the activity of enzymes is improved, and the specificity of substrates and products and the modification of regulatory elements have been changed (Xu et al. 2020; Huang et al. 2020; Das et al. 2020a). The intracellular redox state can be changed by cofactor engineering, energy metabolism can be regulated, carbon flow can be controlled, and the balance of NAD (P) H/NAD (P) ^+^ or ATP/ADP ratio can be carefully monitored to achieve cell redox balance, thus improving the physiological state of the cell factory (Chen et al. 2022b; Yu et al. 2022). At the level of metabolites, structural biotechnology, partition engineering, and modular approach engineering have been reported as common strategies (Chen et al. 2018). After the enzyme is effectively located by structural biotechnology, the concentration of local intermediates in the required biochemical reactions is usually increased, or the damage of intermediates—due to toxicity—is prevented. The advantages of distribution engineering are mainly reflected in concentrating substrates and enzymes, isolating the toxicity of intermediates in the pathway, bypassing inhibitory regulatory networks, and avoiding competition. Modular pathway engineering is mainly used to rebuild metabolic balance and improve metabolite production. Genome-scale engineering and multi-genome editing are the main engineering strategies at the genome level, while cell-level engineering strategies mainly include transporter engineering, morphological engineering, and consortium engineering (Chen et al. 2018; Bharathiraja et al. 2022; Hoek and Borodina 2020; Huo et al. 2020; Duncker et al. 2021). The production of fatty acid derivatives in microbial cell factories is usually regulated by multiple factors rather than by a single factor. Genome-level engineering is mainly used for gene expression intensity regulation, gene integration, gene knockout, and so on, which can maintain the dynamic balance of metabolic pathways and achieve the goal of improving the ability of product synthesis (Liu et al. 2022b; Vuong et al. 2022). Cell-level engineering can reduce feedback inhibition and cytotoxicity, promote balance and downstream reactions, transform microbial cell morphology-related proteins, and purposefully regulate microbial cell morphology and division mode to optimize the characteristics of microbial cells, thus achieving the goal of reducing the cost of biological refining (Chen et al. 2018; Sanchez et al. 2022; Mhatre et al. 2022).

## Challenges and strategies of industrial application

### Challenges

Although we discussed the great potential of the tools of synthetic biology in the previous section, and the application of synthetic biology technology in optimizing the metabolic pathway of microbial cell factories has become more and more mature, using microbial cell factories as a means to capture and fix CO_2_ is still in the "proof-of-concept" stage, and industrial applications still face many challenges (Zhou et al. 2018; Liang et al. 2020a; Keasling et al. 2021). The main challenges of producing FAEE in microbial cell factories can be discussed from both engineering and economic aspects. For example, a review by Zhou et al. (2018) pointed out that it would cost $5000 to $100 m to develop a new cell factory. From laboratory to industrial application, a lot of engineering research is needed to verify and improve production technology, which requires the investment of government policies and social funds to solve this problem. Another challenge is to solve the problem of labor costs, because so far, carbon capture and the use of CO_2_ to obtain high-value chemicals are still two separate industrial processes (Bhatia et al. 2019). In terms of technology, it is necessary to improve the tolerance of microorganisms to CO_2_ to face the high partial pressure of CO_2_ in the flue gas. the high temperature of exhaust gas and the existence of toxic SO_x_ in the flue gas are also the main challenges for the industrial application of CO_2_ capture by microbial cell workers (Patrón and Ricardez-Sandoval 2022; Liang et al. 2020a; Morais et al. 2019; Cheah et al. 2016). Although up to now, it has been reported that the tolerance of microorganisms (e.g., CO_2_ tolerance, high-temperature tolerance, and SO_x_ toxicity tolerance) has been improved in the laboratory through synthetic biology, it takes a lot of effort to be used in industry (Choi et al. 2021). In addition, the selection of the ideal host, the determination of metabolic pathway, and the determination of efficient enzymes will make it difficult to build microbial cell factories in the laboratory, let alone in actual industrial production (Keasling et al. 2021; Morais et al. 2019).

### Photoelectric–microbial coupling system for producing fatty acid derivatives

As mentioned in the previous section, synthetic organisms make it possible to solve the technical challenges of microbial cell factories for the production of biofuels and industrial applications by optimizing metabolic pathways. However, the process of microbial metabolism is accompanied by low biosynthesis efficiency and complex by-product production. at the same time, the energy demand of microbial cell factories is also an obstacle to industrial application. Chemical approaches such as photosynthesis and electrolysis are usually used to convert zero-energy-density CO_2_ into high-energy-density FAs and their derivatives. For example, CO_2_ can be converted into methanol and other liquid fuels by photocatalysis using sunlight as energy (Wu et al. 2019). Similarly, under the action of the catalyst, using electric energy as the energy source, CO_2_ is reduced to valuable compounds, such as formic acid (Ma et al. 2019). However, challenges such as high energy consumption, hazardous catalysts, and high substrate purity make it difficult to carry out efficiently in the conversion process only by chemical methods (Munkajohnpong et al. 2020; Salehizadeh et al. 2020; Woo, 2017). The emergence of an optically coupled microbial system provides a potential new solution to solve these problems (Sullivan et al. 2021; Zhuang et al. 2022a; Yang et al. 2022). For example, Bai et al. (2021). modified a bacterium called Rhodopseudomonas palustris to produce biofuels using only three renewable, naturally rich raw materials (carbon dioxide, electricity, and light from solar panels). Li et al. (2021) reported an integrated photoelectrochemical structure, in which electrons are transferred directly to the photosynthetic electron transfer chain in living cyanobacteria and constructed an artificial photosynthetic system for the production of biofuels. Of course, these technologies are still in their infancy, and more efforts are needed for industrial applications.

As mentioned above, so far, CO_2_ capture and conversion are still two separate processes. Plants and photosynthetic microorganisms can capture and fix CO_2_ directly from the environment, which is a natural integrated system of capture and production, which provides an idea for the establishment of integrated carbon capture and utilization system. Using synthetic organisms as a technical means to construct tolerant bacteria can adapt to extreme environments (e.g., flue gas), and further combined with photoelectrochemical technology to build an integrated system of CO_2_ capture and FAEE production has a great prospect (Bhatia et al. 2019; Sullivan et al. 2021; Izadi and Harnisch, 2022).

## Conclusions

Converting captured CO_2_ into fatty acid derivatives is an attractive solution to climate change (Greenhouse Effect). A microbial cell factory as a means of capturing CO_2_ and converting it into fatty acid derivatives is a promising method. By constructing or optimizing the biosynthesis pathways of endogenous ethanol, FAs, FAEE, etc., engineering microorganisms can directly convert CO_2_ into FAs-derived biofuels. However, up to now, the technology is still in the initial technical stage and can not meet the actual industrial application. In terms of engineering technology, through synthetic biology, we can effectively construct microbial cell factories and optimize their metabolic pathways, and obtain engineering bacteria with more powerful enzyme systems and tolerance to the harsh environment (e.g., high temperature, and flue gas toxicity) for CO_2_ capture and fixation. At the same time, the optoelectronic–microbial hybrid coupling system can accelerate the industrial application process of fatty acid derivatives production in microbial cell factories and is even expected to build an efficient system for integrated carbon capture and utilization, overcome many challenges in this field (e.g., CO_2_ transport and storage), and improve economic efficiency.

## Data Availability

The data sets used and/or analyzed during the current study are available from the corresponding author upon reasonable request.
